# Supplementary material for: Generalized estimating equation modeling on correlated microbiome sequencing data with longitudinal measures
Source: PLoS Comput Biol. 2020 Sep 8;16(9):e1008108. doi: 10.1371/journal.pcbi.1008108 (PMC7500673; doi:10.1371/journal.pcbi.1008108)
Supplement: S1 Appendix — (PDF) [file pcbi.1008108.s002.pdf]

Generalized Estimating Equation Modeling on Correlated Microbiome Sequencing Data with Longitudinal Measures

Bo Chen[1], Wei Xu[1,2*],

1 Princess Margaret Hospital, 610 University Avenue, Toronto, Ontario, Canada

2 Dalla Lana School of Public Health, University of Toronto, Toronto, Ontario, Canada

* wei.xu@uhnresearch.ca

Appendix 1: Simulation from (generalized) linear mixed models

In section "Simulation settings", we simulated $\boldsymbol{Y}^{(0)}$ and $\boldsymbol{Z}$ based on correlation structure $\boldsymbol{R}$. As a sensitivity analysis, here we also simulate $\boldsymbol{Y}^{(0)}$ from generalized linear mixed model and $\boldsymbol{Z}$ from linear mixed model, i.e., independent samples plus random intercepts. We added two random intercepts in each model, one representing OTU correlation and the other representing repeated longitudinal measures. Results are summarized in Table A1 below.

In Table 2, test powers highly depends on the true correlation coefficients in $\boldsymbol{R}$. When simulating under (generalized) linear mixed models, $\boldsymbol{R}$ cannot be explicitly specified, so powers in Table A1 are not directly comparable to the powers in Table 2. We changed true $\hat{\beta}^{(+)}$ value to 0.1 and -0.1 to achieve moderate powers more similar to Table 2. Despite the different parameter settings, we find our MTLC model has consistent performance with Table 2. We also observed a small type I error inflation rate under 2P_ind and 1P_ind model.

Table A1: Estimated $\hat{\beta}$, Type I error and power, from 1000 replications, OTU samples simulated under (generalized) linear mixed models

$(\beta^{(0)}, \beta^{(+)})$	Estimates	GEE$^{(0)}$	GEE$^{(+)}$	MTLC	2P_ind	1P_GEE	1P_ind	1P_RE
(0,0)	$\hat{\beta}$	NA	NA	NA	NA	0.001	0.001	0.001
	$\hat{\beta}^{(0)}$	0.001	NA	0.001	0.001	NA	NA	NA
	$\hat{\beta}^{(+)}$	NA	0.001	0.001	0.001	NA	NA	NA
	T1E	0.057	0.038	0.050	0.074	0.040	0.074	0.039
(0,0.1)	$\hat{\beta}$	NA	NA	NA	NA	0.050	0.050	0.050
	$\hat{\beta}^{(0)}$	0.001	NA	0.001	0.001	NA	NA	NA
	$\hat{\beta}^{(+)}$	NA	0.099	0.099	0.099	NA	NA	NA
	Power	0.051	0.605	0.496	0.603	0.440	0.509	0.439
(0,-0.1)	$\hat{\beta}$	NA	NA	NA	NA	-0.051	-0.051	-0.051
	$\hat{\beta}^{(0)}$	-0.002	NA	-0.002	-0.002	NA	NA	NA
	$\hat{\beta}^{(+)}$	NA	-0.099	-0.099	-0.100	NA	NA	NA
	Power	0.056	0.615	0.515	0.626	0.447	0.524	0.442
(0.1,0)	$\hat{\beta}$	NA	NA	NA	NA	0.048	0.048	0.048
	$\hat{\beta}^{(0)}$	0.096	NA	0.096	0.096	NA	NA	NA
	$\hat{\beta}^{(+)}$	NA	-0.001	-0.001	-0.001	NA	NA	NA
	Power	0.858	0.040	0.790	0.808	0.418	0.485	0.418
(0.1,0.1)	$\hat{\beta}$	NA	NA	NA	NA	0.099	0.099	0.099
	$\hat{\beta}^{(0)}$	0.099	NA	0.099	0.099	NA	NA	NA
	$\hat{\beta}^{(+)}$	NA	0.100	0.100	0.100	NA	NA	NA
	Power	0.871	0.616	0.923	0.951	0.952	0.962	0.953
(0.1,-0.1)	$\hat{\beta}$	NA	NA	NA	NA	0.000	0.000	0.000
	$\hat{\beta}^{(0)}$	0.099	NA	0.099	0.099	NA	NA	NA
	$\hat{\beta}^{(+)}$	NA	-0.100	-0.100	-0.099	NA	NA	NA
	Power	0.872	0.614	0.934	0.945	0.055	0.086	0.055
(-0.1,0)	$\hat{\beta}$	NA	NA	NA	NA	-0.048	-0.048	-0.048
	$\hat{\beta}^{(0)}$	-0.099	NA	-0.099	-0.099	NA	NA	NA
	$\hat{\beta}^{(+)}$	NA	0.003	0.003	0.003	NA	NA	NA
	Power	0.870	0.047	0.803	0.814	0.415	0.477	0.417
(-0.1,0.1)	$\hat{\beta}$	NA	NA	NA	NA	0.002	0.002	0.002
	$\hat{\beta}^{(0)}$	-0.098	NA	-0.098	-0.098	NA	NA	NA
	$\hat{\beta}^{(+)}$	NA	0.103	0.103	0.103	NA	NA	NA
	Power	0.866	0.641	0.929	0.942	0.057	0.080	0.053
(-0.1,-0.1)	$\hat{\beta}$	NA	NA	NA	NA	-0.098	-0.098	-0.098
	$\hat{\beta}^{(0)}$	-0.098	NA	-0.098	-0.098	NA	NA	NA
	$\hat{\beta}^{(+)}$	NA	-0.099	-0.099	-0.099	NA	NA	NA
	Power	0.869	0.645	0.930	0.944	0.951	0.965	0.950

Appendix 2: Empirical distributions of $\hat{\beta}$

We show the 2.5 and 97.5 percentile of the empirical distributions of $\hat{\beta}$ in Table 2 in Table A2 below.

Table A2: 2.5 and 97.5 percentile of the empirical distributions of $\hat{\beta}$

$(\beta^{(0)}, \beta^{(+)})$	Estimates	GEE$^{(0)}$	GEE$^{(+)}$	MTLC	2P_ind	1P_GEE	1P_ind	1P_RE
(0,0)	$\hat{\beta}$	NA	NA	NA	NA	(-0.045,0.047)	(-0.045,0.047)	(-0.045,0.047)
	$\hat{\beta}^{(0)}$	(-0.071,0.083)	NA	(-0.071,0.083)	(-0.071,0.083)	NA	NA	NA
	$\hat{\beta}^{(+)}$	NA	(-0.049,0.048)	(-0.049,0.048)	(-0.048,0.049)	NA	NA	NA
(0,0.05)	$\hat{\beta}$	NA	NA	NA	NA	(-0.017,0.071)	(-0.017,0.071)	(-0.017,0.071)
	$\hat{\beta}^{(0)}$	(-0.075,0.078)	NA	(-0.075,0.078)	(-0.075,0.078)	NA	NA	NA
	$\hat{\beta}^{(+)}$	NA	(0.002,0.098)	(0.002,0.098)	(-0.001,0.098)	NA	NA	NA
(0,-0.05)	$\hat{\beta}$	NA	NA	NA	NA	(-0.074,0.021)	(-0.074,0.021)	(-0.074,0.021)
	$\hat{\beta}^{(0)}$	(-0.084,0.074)	NA	(-0.084,0.074)	(-0.084,0.074)	NA	NA	NA
	$\hat{\beta}^{(+)}$	NA	(-0.101,0.000)	(-0.101,0.000)	(-0.102,0.003)	NA	NA	NA
(0.1,0)	$\hat{\beta}$	NA	NA	NA	NA	(0.004,0.097)	(0.004,0.097)	(0.004,0.097)
	$\hat{\beta}^{(0)}$	(0.020,0.178)	NA	(0.020,0.178)	(0.020,0.178)	NA	NA	NA
	$\hat{\beta}^{(+)}$	NA	(-0.05,0.051)	(-0.05,0.051)	(-0.053,0.053)	NA	NA	NA
(0.1,0.05)	$\hat{\beta}$	NA	NA	NA	NA	(0.028,0.122)	(0.028,0.122)	(0.028,0.122)
	$\hat{\beta}^{(0)}$	(0.021,0.178)	NA	(0.021,0.178)	(0.021,0.178)	NA	NA	NA
	$\hat{\beta}^{(+)}$	NA	(0.000,0.101)	(0.000,0.101)	(0.001,0.104)	NA	NA	NA
(0.1,-0.05)	$\hat{\beta}$	NA	NA	NA	NA	(-0.022,0.073)	(-0.022,0.073)	(-0.022,0.073)
	$\hat{\beta}^{(0)}$	(0.022,0.181)	NA	(0.022,0.181)	(0.022,0.181)	NA	NA	NA
	$\hat{\beta}^{(+)}$	NA	(-0.102,-0.002)	(-0.102,-0.002)	(-0.101,0.000)	NA	NA	NA
(-0.1,0)	$\hat{\beta}$	NA	NA	NA	NA	(-0.097,-0.002)	(-0.097,-0.002)	(-0.097,-0.002)
	$\hat{\beta}^{(0)}$	(-0.186,-0.019)	NA	(-0.186,-0.019)	(-0.186,-0.019)	NA	NA	NA
	$\hat{\beta}^{(+)}$	NA	(-0.055,0.050)	(-0.055,0.050)	(-0.055,0.051)	NA	NA	NA
(-0.1,0.05)	$\hat{\beta}$	NA	NA	NA	NA	(-0.070,0.022)	(-0.070,0.022)	(-0.070,0.022)
	$\hat{\beta}^{(0)}$	(-0.175,-0.022)	NA	(-0.175,-0.022)	(-0.175,-0.022)	NA	NA	NA
	$\hat{\beta}^{(+)}$	NA	(0.001,0.100)	(0.001,0.100)	(-0.001,0.102)	NA	NA	NA
(-0.1,-0.05)	$\hat{\beta}$	NA	NA	NA	NA	(-0.120,-0.029)	(-0.120,-0.029)	(-0.120,-0.029)
	$\hat{\beta}^{(0)}$	(-0.177,-0.024)	NA	(-0.177,-0.024)	(-0.177,-0.024)	NA	NA	NA
	$\hat{\beta}^{(+)}$	NA	(-0.101,0.002)	(-0.101,0.002)	(-0.103,0.006)	NA	NA	NA
