# Supplementary material for: Generalized estimating equation modeling on correlated microbiome sequencing data with longitudinal measures
Source: PLoS Comput Biol. 2020 Sep 8;16(9):e1008108. doi: 10.1371/journal.pcbi.1008108 (PMC7500673; doi:10.1371/journal.pcbi.1008108)
Supplement: Attachment — Submitted filename: PLOSreview.pdf [file pcbi.1008108.s003.pdf]

In this article, the authors present a novel method to analyze microbiome data for disease prediction and correlation analysis. It is very important to build a statistical and computational model which fully accounts for the correlation relationships among the OTUs.

The theoretical investigations are rigorous and the numeric studies provide very comprehensive assessment of the empirical performance of the proposed methods. The paper is well organized and all the theoretical and numerical results are presented in a very concise and rigorous way.

The authors proposed to use a two-part Microbiome Taxonomic Longitudinal Correlation (MTLC) model for multivariate zero-inflated OTU outcomes based on the GEE framework. Longitudinal and other types of repeated OTU measures are integrated in the MTLC model. Variance estimators of the proposed regression estimates are fully developed. Compared with the existing methods including traditional GEE and mixed models, the MTLC method is shown to be more powerful and more accurate in numerical studies. Authors have also investigated the performance of the method on a real microbiome data.

Compared to existing predictive methods, the newly proposed method is advantageous for microbiome data analysis because it models the correlation structure among the repeated measurements and among the correlated OTUs. The authors have provided novel and significant contributions in this paper. I strongly recommend the publication of this paper.

A minor comment is regarding the dimension of the predictor. It would be helpful to the readers if the authors can discuss how the model can be extended to accommodate higher dimensional predictors which often arises in practical applications.
