# Supplementary material for: Generalized estimating equation modeling on correlated microbiome sequencing data with longitudinal measures
Source: PLoS Comput Biol. 2020 Sep 8;16(9):e1008108. doi: 10.1371/journal.pcbi.1008108 (PMC7500673; doi:10.1371/journal.pcbi.1008108)
Supplement: Attachment — Submitted filename: Responses.pdf [file pcbi.1008108.s004.pdf]

Response to **"Generalized Estimating Equation Modeling on Correlated Microbiome Sequencing Data with Longitudinal Measures"**

Bo Chen[1], Wei Xu[1,2*],

1 Princess Margaret Hospital, 610 University Avenue, Toronto, Ontario, Canada

2 Dalla Lana School of Public Health, University of Toronto, Toronto, Ontario, Canada

* wei.xu@uhnresearch.ca

We would like to thank the reviewers for the valuable suggestions and comments which have significantly improved the content and presentation of the paper.

Reviewer #1:

In this article, the authors present an innovative method to analyze microbiome data for disease prediction and correlation analysis. It is very important to build a statistical and computational model which fully accounts for the correlation relationships among the OTUs.
The theoretical investigations are rigorous and the numeric studies provide very comprehensive assessment of the empirical performance of the proposed methods. The paper is well organized and all the theoretical and numerical results are presented in a very concise and rigorous way.
The authors proposed to use a two-part Microbiome Taxonomic Longitudinal Correlation (MTLC) model for multivariate zero-inflated OTU outcomes based on the GEE framework. Longitudinal and other types of repeated OTU measures are integrated in the MTLC model. Variance estimators of the proposed regression estimates are fully developed. Compared with the existing methods including traditional GEE and mixed models, the MTLC method is shown to be more powerful and more accurate in numerical studies. Authors have also investigated the performance of the method on a real microbiome data.
Compared to existing predictive methods, the newly proposed method is advantageous for microbiome data analysis because it models the correlation structure among the repeated measurements and among the correlated OTUs. The authors have provided innovative and significant contributions in this paper. I strongly recommend the publication of this paper.

Response: We thank the reviewer for the encouraging comments on our manuscript.

A minor comment is regarding the dimension of the predictor. It would be helpful to the readers if the authors can discuss how the model can be extended to accommodate higher dimensional predictors which often arises in practical applications.

Response: Thanks for the comment. We agree with the reviewer that it would be helpful to discuss the possible extension of our model to accommodate high dimensional predictors, and we have added the following paragraph in discussion section to briefly discuss the high dimensional predictors situation.

"For the proposed method, the dimension of predictors' design matrix $\boldsymbol{X_k}$, p, is assumed to be less than the number of clusters K. For high dimensional predictor space, e,g., gene expressions in genome-wide association study, it is possible to encounter the situation of $p \geq K$. In such cases regression models cannot be directly applied, and dimension reduction techniques need to be used. Traditional approaches such as principal component analysis and penalized regression including ridge regression and LASSO, as well as some machine learning based feature selection methods can be considered to be incorporated into the proposed method to deal with high dimensional predictors. We are planning to extend the proposed method to deal with such high dimensional predictors situation."

Reviewer #2:

In this paper, the authors proposed a Microbiome Taxonomic Longitudinal Correlation (MTLC) model to test the association between operational taxonomic units (OTUs) and the predictors of interest. The model consists of two parts: a logistic regression of the OTU prevalence and a linear regression of OTU relative abundance (RA). An omnibus test of these two regressions was developed to assess overall statistical significance. The model parameters and their variances are estimated by Generalized Estimating Equation (GEE) approach to account for the correlation between the OTUs and repeated measures. The simulation study shows that the proposed method can control type I error at the nominal level 0.05 and the power is the most robust against different configurations of the effect sizes. The advantage of the proposed method is further evidenced by a real data analysis in which the association between obesity and OTUs was detected by the proposed method but not by other comparative methods.
The idea of applying GEE to model longitudinal/correlated data is not new

and but a two-part GEE with estimated correlation matrix seems to be novel in microbiome studies. However, some major concerns about the proposed statistical model, the simulation settings and the power comparison results need to be addressed.

Response: We appreciate the reviewer's comments. We have addressed specific comments related to the proposed statistical model, the simulation and the power comparison results in the following point by point response.

General:

1. The authors mention in the Abstract and Introduction that the proposed method is able estimate the correlations between OTUs. However, the benefits of obtaining those correlation coefficients are not clearly stated. What additional information can an accurate estimated correlation bring to us?

Response: Thanks for the comment. We agree with the reviewer that the benefits of obtaining accurate correlation estimation should be clarified. We have added the following sentences in introduction section to clearly state the benefits of correlation estimation.

"Identifying correlations between taxa is a common goal in genomic survey (Friedman & Alm, 2012). An accurate estimated correlation can be used to determine drivers in environmental ecology or contribution to habitat niches or disease; it is also a powerful tool to help researchers with hypothesis generation, such as determining which interactions might be biologically relevant in their system, and should be given further study (Weiss *et al.*, 2016)."

2. The presented bibliography is rich. However, previous applications of GEE in microbiome data were not mentioned, e.g.
Kelly, B.J., Imai, I., Bittinger, K. et al. Microbiome 4, 7 (2016). https://doi.org/10.1186/s40168-016-0151-8
Seekatz, A.M., Rao, K., Santhosh, K. et al. Genome Med 8, 47 (2016). https://doi.org/10.1186/s13073-016-0298-8.

Response: Thanks for pointing it out. We agree with the reviewer that applications of GEE in microbiome data should also be referenced, in addition to GEE application in longitudinal zero-inflated data. We have added these papers in the introduction section of the revised manuscript.

Statistical Model:

1. The statistical model is confusing. The authors seem to assume that, within each independent block, y and x are linked by a GLM (1). The y0, i.e. the dichotomized y, and y+, i.e. the truncated y>0, relate to x by other GLMs (5) and (6), respectively. If so, the distribution of y can be and should

be stated explicitly. If not, what is the relationship between y and (y0, y+)? This also concerns the data generating distribution in the simulation. See the comments regarding simulations.

Response: Thanks for the comment. We made some clarifications in the methodology section (Page 9) and the simulation section (Page 11) in the revised manuscript to make it clear. The distributions of zero-inflated log normal samples $\boldsymbol{Y}$, its two parts and their relationships are now all clearly stated by mathematical formulas. To avoid any confusion, we also explained that the two parts $\boldsymbol{Y}^{\mathbf{0}}$ and $\boldsymbol{Y}^{+}$, rather than $\boldsymbol{Y}$, are directly related to $\boldsymbol{X}$ via GEE model.

2. The authors claim that the test statistics W0 and W+ are independent (P.9 line 211). I am not convinced this is true for arbitrary y as it seems to be presented in the paper. In fact, given a vector y, the number of non-zero elements, i.e. E(y0), determines the length of y+ which seems to contradict the independence claim. The authors should clarify under what kind of distribution of y this independence property holds.

Response: We appreciate this critical comment regarding our statistical model. In the original manuscript, what we were trying to show was conditional independence between $\boldsymbol{Y}^{+}|\boldsymbol{Y}^{\mathbf{0}}$ and $\boldsymbol{Y}^{\mathbf{0}}$. However, we realized that our proof may be problematic, as there lacks of rigorous definition in probability theory for conditional random variable. The general independence between $\boldsymbol{Y}^{+}$ and $\boldsymbol{Y}^{\mathbf{0}}$, as you mentioned, may not be true. Therefore, we have changed the original combined Chi-squared test to Cauchy combination test (CCT), which does not require the two parts to be independent. In the revised manuscript, we applied CCT and removed the chi-squared test in our simulation and application studies, and we have updated results in Table 2, 4 and 5 and relevant texts.

3. An omnibus test W_MTLC (7) is proposed to combine W0 and W+ by summation. Other approaches can be used to combine the p-values of these two tests, e.g. the minimum P-value (minP) approach and Cauchy combination test (CCT) Yaowu Liu & Jun Xie (2020) JASA, 115:529, 393-402, DOI: 10.1080/01621459.2018.1554485. Notice that the power of W0 and W+ can be drastically different in the simulations, I would expect the power of either minP or CCT to be higher than the summation of statistics as proposed.

Response: We really appreciate the reviewer's insightful suggestion on Cauchy combination test (CCT). As explained in Comment 2, the CCT is an appropriate test for our MTLC model without the stringent independent assumption. We conducted some additional comparison to compare the power performance (results shown below). between

the original Chi-squared test and CCT, and found both tests have very similar performance. We also explored minP approach but decided to choose CCT, because minP can be very time consuming to find its empirical distribution using permutation, while the underlying distribution of CCT has clear theoretical justification and computational benefits.

(β_B, β_N)	GEE logis	GEE+	$\chi^2_{(2)}$	CCT
(0,0)	0.056	0.038	0.043	0.039
(0,0.05)	0.045	0.512	0.417	0.421
(0,-0.05)	0.048	0.487	0.386	0.394
(0.1,0)	0.693	0.050	0.596	0.609
(0.1,0.05)	0.705	0.487	0.794	0.771
(0.1,-0.05)	0.696	0.481	0.800	0.764
(-0.1,0)	0.700	0.054	0.604	0.612
(-0.1,0.05)	0.719	0.483	0.825	0.803
(-0.1,-0.05)	0.694	0.471	0.820	0.786

Simulation:

1. The data generating distribution/process (P.9 lines 239-246 and P.9 lines 252-256) should be clearly written in statistical symbols/equations to avoid confusion. Most importantly, how are Y0, Y+ and Y related to the predictor X?

Response: Thanks for pointing it out. We have made the change on Page 11, and clearly stated the distribution of $\boldsymbol{Y}^{(0)}$ and $\boldsymbol{Z}$ as functions of $\boldsymbol{X}$, and $\boldsymbol{Y}$ computed as the product of $\boldsymbol{Y}^{(0)}$ and $\boldsymbol{Z}$.

2. It seems that this simulation setting is fundamentally different from the simulations in literature, e.g. [11], [16], in which the distribution of Y is clearly defined, while in this paper, Y is constructed by Y0 and Y+. It would be very helpful if authors can

2.1. explain why they choose such way to simulate Y0 and Y+ and Y.

Response: Thanks for the comment. We have added the following paragraph on Page 12 in the revised manuscript to clarify the stimulation setting.

"Different from some literature that $\boldsymbol{Y}$ is directly simulated, we conducted our stimulation on $\boldsymbol{Y}^{(0)}$ and $\boldsymbol{Z}$ separately. This is because following the mixture distribution framework, we conduct two separate GEE models on $\boldsymbol{Y}^{(0)}$ and $\boldsymbol{Y}^{(+)}$ rather than one model directly on $\boldsymbol{Y}$. In this way, we can clearly specify the true values of predictor's main effects and OTU correlations in simulation settings, and evaluate if the estimations of these values are unbiased explicitly."

2.2. explain how to interpret the effect sizes beta0 and beta+. Are we particularly interested in detecting a pair of (beta0, beta+) in different

directions (as shown in Table 2, this is where the proposed method has the largest power gain) in real data?

Response: Thanks for the comment. There are some studies which evaluated the opposite directions of $\beta^{(0)}$ and $\beta^{(+)}$ (Lachenbruch, 2001; Xu *et al.*, 2015; Costelloe *et al.*, 2010; Munita & Arias, 2016). We updated the manuscript by adding the following paragraphs in the discussion section.

"When $\beta^{(0)}$ and $\beta^{(+)}$ have opposite signs, the predictor's effects are described as "dissonant". Under this scenario, the two-part models showing more powerful results in the simulation studies coincides with existing literature (Lachenbruch, 2001; Xu *et al.*, 2015). In microbiome context, an example of this scenario is that, an antibiotic treatment may be effective in reducing the risk of carrying some specific bacteria, but may result in the growth of these bacteria once they survive due to antibiotic resistance (Costelloe *et al.*, 2010; Munita & Arias, 2016)."

2.3. simulate Y from a simpler model, e.g. a linear mixed model, as a sensitivity analysis to see if the proposed method still works fine.

Response: Thanks for the reviewer's comment. Following the reviewer's suggestion, we conducted sensitivity analysis by simulated $\boldsymbol{Y}^{(0)}$ and $\boldsymbol{Z}$ under linear mixed model. Our MTLC model showed robust performance under this new simulation. We have added these results into Appendix Table A1 of the revised manuscript.

3. Table 2. Estimated beta, Type I error and power:
3.1. The authors state that when beta0 and beta+ are in the same direction, the two-parts model is still more powerful in "general" (P.13 lines 307-308). This is simply not true. In Table 2, there are only two rows (row 6 and 10) where effects are in the same direction. In both cases, the 1P_GEE and 1P_RE both have higher power than MTLC.

Response: Thanks very much for pointing out this mistake. We removed this sentence from the revised manuscript.

3.2. The authors should make it clear that the estimated beta, beta0, beta+ are from one simulation or the average from 1,000 replication? A more sensible way is to report the empirical distribution of the estimated beta, e.g. standard deviation, 2.5 and 97.5 percentile of the empirical distribution.

Response: Thanks for the comment. We described on Page 12 that the simulation was based on 1000 replications. To make it more clear, we have updated the caption of Table 2 to include this information. Following reviewer's suggestion, we have added the 2.5 and 97.5 percentile of the empirical distribution in Appendix Table A2.

Minor (typos, etc.):
1. Author Summary and P.1 line 2: "...fast-growing...".

2. P.2 lines 5-6: "...shotgun metagenomics sequencing...".

3. P.5 line 106: "...other entries...".

4. P.8 line 185, P11 line 282: "Next, we...".

5. P.9 lines 196-197: mu0 (5) and mu+ (6) are not defined.

6. P.10 line 231: "...may be greater than...".

7. P.12 Table 2: (beta_B, beta_N) should be (beta0, beta+).

8. P.14 line 363: "...one-part models...".

9. P.15 line 375: "... at level genus."

10. P.18 line 441: "...distribution...is unknown..."

Response: Thanks for pointing these out. We have fixed these typos in the revised manuscript accordingly.

References

Costelloe, C., Metcalfe, C., Lovering, A., Mant, D., & Hay, A. D. 2010. Effect of antibiotic prescribing in primary care on antimicrobial resistance in individualpatients: systematic review and meta-analysis. *British Medical Journal*, **340**(7756), 1120.

Friedman, J., & Alm, E. J. 2012. Inferring Correlation Networks from Genomic Survey Data. *PLoS Computational Biology*, **8**(9), e1002687.

Lachenbruch, P. A. 2001. Comparisons of two-part models with competitors. *Statistics in Medicine*, **20**, 1215–1234.

Munita, J. M., & Arias, C. A. 2016. Mechanisms of Antibiotic Resistance. *Microbiology Spectrum*, **4**(2).

Weiss, S., Treuren, W. V., Lozupone, C., Faust, K., Friedman, J., & et al. 2016. Correlation detection strategies in microbial data sets vary widely in sensitivity and precision. *The ISME Journal*, **10**, 1669–1681.

Xu, L., Turpin, W., Paterson, A. D., & Xu, W. 2015. Assessment and Selection of Competing Models for Zero-Inflated Microbiome Data. *PLoS ONE*, **10**(7), e0129606.
